# A simple adeno-associated virus-based approach for the generation of cardiac genetic models in rats

**DOI:** 10.12688/f1000research.27675.1

**Published:** 2020-12-10

**Authors:** Michal Schlesinger-Laufer, Guy Douvdevany, Lilac Haimovich-Caspi, Yaniv Zohar, Rona Shofty, Izhak Kehat

**Affiliations:** 1 The Pre-Clinical Research Authority Unit, The Technion, Israel Institute of Technology, 1 Efron Street, P.O. Box 9697, Haifa, 3109601, Israel; 2 Faculty of Medicine, Technion - Israel Institute of Technology, 1 Efron Street, P.O. Box 9697, Haifa, 3109601, Israel; 3 Department of Pathology, Rambam Medical Center, HaAliya HaShniya St 8, Haifa, 3109601, Israel

**Keywords:** Cardiac research, animal models, Adeno-associated virus

## Abstract

**Background:** Heart failure is a major health problem and progress in this field relies on better understanding of the mechanisms and development of novel therapeutics using animal models. The rat may be preferable to the mouse as a cardiovascular disease model due to its closer physiology to humans and due to its large size that facilitates surgical and monitoring procedures. However, unlike the mouse, genetic manipulation of the rat genome is challenging.

**Methods:** Here we developed a simple, refined, and robust cardiac-specific rat transgenic model based on an adeno-associated virus (AAV) 9 containing a cardiac troponin T promoter. This model uses a single intraperitoneal injection of AAV and does not require special expertise or equipment.

**Results**: We characterize the AAV dose required to achieve a high cardiac specific level of expression of a transgene in the rat heart using a single intraperitoneal injection to neonates. We show that at this AAV dose GFP expression does not result in hypertrophy, a change in cardiac function or other evidence for toxicity.

**Conclusions:** The model shown here allows easy and fast transgenic based disease modeling of cardiovascular disease in the rat heart, and can also potentially be expanded to deliver Cas9 and gRNAs or to deliver small hairpin (sh)RNAs to also achieve gene knockouts and knockdown in the rat heart.

## Abbreviations

Adeno-Associated Virus (AAV); Viral genomes (vg); Short hairpin RNAs (shRNA); Fractional shortening (FS%); left ventricular end diastolic dimeter (LVIDd); enhanced green fluorescent protein (eGFP); cardiac troponin T (cTnT)

## Introduction

Cardiovascular disease is the leading global cause of mortality and is expected to account for >22.2 million deaths by 2030 ^1^. Despite some progress in the medical treatment of heart failure, the 5-year mortality is still dismal, at about 50%, a worse prognosis than in most cancers. With the continued aging of the population, an increase in the number of new patients is estimated. Unless there is substantial progress in prevention and treatment, heart failure will remain a major health problem ^2^.

Continuous progress in therapy relies on a better understanding of the pathobiology of heart failure, and studies to discover new mechanisms and test new therapeutics are direly needed ^3^. Animal modeling of cardiovascular disease is challenging, and most models aim to create the etiological factors leading to cardiac disease either by surgery, selective breeding, or genetic modifications. Small animal models, particularly mice and rats, are essential models in cardiac research allowing for relatively rapid, high through-put, and cost effective means of studying cardiac physiology, disease, and novel therapeutic targets ^4^.

The rat has several advantages over the mouse for cardiovascular research. It offers some of the advantages of a larger animal but with reduced costs, and it is preferable for surgical procedures. Technically it is more feasible to create ischemia, pressure, or volume overload models by coronary artery ligation, aortic banding, or shunt procedures respectively in the rat than in the mouse ^5^, and those models are well established in the literature ^6^. It is easier to perform physiological monitoring in the rat, and in many cases, the physiology is more similar to humans ^7^. Rats also have a greater ability to increase their heart rate during exercise, have a more positive FFR (force-frequency relationship) and have slightly slower kinetics of contraction and relaxation as compared to mice ^8^. Indeed, the rat became the initial small animal model of choice for cardiovascular research ^9^.

Functional genomics aimed at studying the effects of changes in gene expressions by targeted mutagenesis or transgenesis allowed many insights into signaling pathways involved in the pathogenesis of heart failure ^10^. With the sequencing of the mouse genome and the development of many genetically modified mouse lines in the last 20 years, the mouse has become widely used as a tool in generating complex myocardial phenotypes ^11^. However, functional genomic research in the rat has been limited due to difficulties in manipulation of the rats genome ^7^. As a result, despite being the most remote rodent from humans in terms of contractile function, the mouse became the most used animal model for cardiovascular research.

Here we aimed to develop a simplified cardiac-specific rat transgenic model based on a single adeno-associated virus (AAV) injection. We show that we can achieve a robust high level and specific cardiac expression of transgene in the rat heart. This model will allow easy and fast transgenic based disease modeling of cardiovascular disease in the rat heart.

## Methods

### Animals

A total of 15 HsdHan: Wistar rat pups were used in this study. Sample size of n=5 per group was calculated based on preliminary mice experiments with 80% power to detect 1.5-fold increase in mean GFP fluorescence. All pups were born at the SPF unit of the pre-clinical research authority at the Technion (Israel Institute of Technology; IIT). Health monitoring was carried out in accordance with FELASA recommendations ^12^. Each litter was housed with the dam, weened at 3 weeks and separated by sex. Rats were group housed in IVC racks in Sealsafe Plus GR900 TECNIPLAST cages, on Sani chips bedding (Teklad ENVIGO) and Tek-fresh bedding (Teklad ENVIGO). Disposable play tunnels were added as environmental enrichment. Rats were maintained under climate-controlled conditions of 12:12 hrs light/dark cycle, temperature range 21±2°C, a relative humidity of 30–70% and fed *ad libitum* a commercial food – pellet diet (Altromin 1414 IRR). *Ad libitum* reverse osmosis acidified water (pH of 3.0±0.2) was accessible in polycarbonate water bottles covered with stainless steel lids. All efforts were made to ameliorate any suffering of animals, and treatment consisted of a single intraperitoneal injection, anesthesia during echocardiography, and euthanasia at the end of protocol. Daily monitoring by a veterinarian ensured animal well-being.

Studies were conducted at the Technion (IIT), Faculty of Medicine, Haifa, Israel, after obtaining approval from the institute’s IACUC. All proceedings complied with the Animal Welfare Act of 1966 (P.L. 89-544), as amended by the Animal Welfare Act of 1970 (P.L.91-579) and 1976 (P.L. 94-279) *.* Animals allocation to control and experimental groups was done randomly. Cages were arranged on the racks by a technician unaware of the experimental plan. Cages were then allocated to the control, ‘low dose’, and ‘high dose’ groups according to their order on the rack without prior examination of the rats in the cages to avoid bias. Viral injection, echocardiography, euthanasia, and sample analysis were each done on all the animals in the same day to minimize confounders. Echocardiography and histology (H&E) analyses were performed by an investigator unaware of the group allocation of the animals. Because the GFP fluorescence was obvious, the investigator performing the fluorescence microscopy could not be blinded to the treatment. To avoid bias in the GFP fluorescence analysis, we performed and show analysis of the entire heart sections. High power fields were picked at random without looking at the green fluorescence.

### AAV production

The following plasmids were used for AAV production: pENN.AAV.cTNT.PI.eGFP.WPRE.rBG was a gift from James M. Wilson ( Addgene plasmid #105543; RRID:Addgene_105543); pAdDeltaF6 was a gift from James M. Wilson ( Addgene plasmid #112867; RRID:Addgene_112867); pAAV2/9n was a gift from James M. Wilson ( Addgene plasmid #112865; RRID:Addgene_112865).

AAV was produced as previously described in detail ^13^. In brief, ten 150 mm dishes of HEK293T cells (ATCC) were triple transfected using polyethylenimine (PEI). Media containing virus was collected after 72 hours and combined with the media and lysate that were collected after 120 hours. Both lysate and media were purified over iodixanol gradient (Optiprep, Sigma D1556-250ML) via ultracentrifugation. Buffer was exchanged to PBS via amicons (Millipore, UFC910008). AAV titration was conducted to determine viral particle load by qPCR with AAV transfer plasmid as a positive control to create a standard curve using WPRE primers.

### AAV injection

We used two viral stock concentrations - a ‘low dose’ of 4×10 ^12^ viral genomes (vg)/ml, and a ‘high dose’ of 2.6×10 ^13^ viral genomes (vg)/ml concentrations. Five-day-old rat pups (n=5 pups per group) received a single 50 µl intra-peritoneal injection of saline (control group), 2×10 ^11^ viral genomes (low dose), or 1.3×10 ^12^ viral genomes (high dose) using a 30-gauge needle. Injections were performed in all the animals in the morning at the animal facility. Rats were maintained in house and analyzed at 12 weeks of age.

### Gravimetry

After euthanasia the animal weight was measured on a laboratory scale (Precisa BJ 610C). The heart was harvested, washed with cold PBS, and blotted on Kimwipe tissue paper, and the weight measured on a laboratory scale (Precisa XT 220A).

### Echocardiography

At 12 weeks of age, rats were anesthetized with 2% Isoflurane; body temperature was maintained by placing the rats on a warm 40°C heating plate. Breathing and heart rate were monitored throughout the procedure. Echocardiography was performed using a High-Resolution Ultrasound Imaging system Vevo2100 (Visual Sonics, Fujifilm) using a MS 250 13–24 MHz linear array transducer. Measurements were performed on parasternal short axis view at the level of the papillary muscles using M-Mode. Fractional shortening (FS%) was calculated as follows: FS (%) = [(LVIDd − LVIDs) /LVIDd] × 100. All values were based on the median of 3 independent measurements for each rat.

### Histology and immunofluorescence

Hearts were fixed in ice cold 4% formaldehyde in PBS for 2 hours, then placed in cryopreservation solution containing 30% sucrose in PBS at 4°C overnight. The next day hearts were washed in cold PBS, embedded in optimal cutting temperature compound and snap frozen in 2-methylbutane immersed in liquid nitrogen. Sectioning of the hearts was performed in a cryostat (Leica) at 5 µm intervals. Hematoxylin and eosin (H&E) staining was performed using standard protocols and imaged with 3DHistech Pannoramic 250 Flash III automatic slide scanner. For immunofluorescence, cardiac sections were permeabilized with 1% Triton and blocked with a solution containing 5% bovine serum. Primary antibody monoclonal Anti-sarcomeric-alpha-Actinin (catalog number A7811, clone EA-53, Sigma-Aldrich) was incubated overnight at 4°C. Secondary antibody (Jackson ImmunoResearch, catalog number 715-175-151) was incubated for 1 hour at room temperature. Nuclei were counterstained with DAPI for 10 min at room temperature. Slides were imaged with Axio Observer inverted fluorescent microscope (Zeiss) using an X-cite metal-halide light source and a high-resolution camera (Hamamatsu Orca R2) and with 3DHistech Pannoramic 250 Flash III slide scanner.

### RNA extraction, reverse transcription, and quantitative real time PCR (qRT-PCR)

RNA was purified from apical segments of hearts using TRI-Reagent (Sigma-Aldrich), according to the manufacturer's protocol. RNA was then reverse transcribed with 5x All-In-One Reverse Transcriptase MasterMix (Applied Biological Materials, Inc). Quantitative real‐time PCR was performed with iTaq universal SYBR green supermix (Bio‐Rad) using Bio‐Rad CFX96 real‐time system (model C100 Touch). Cycling conditions were: step 1 - 95°C for 3 minutes, step 2 - 95°C for 10 sec, step 3 - 55°C for 30 sec and read plate. Steps 2–3 were repeated 39 times. Expression data were normalized to the expression of Gapdh and ribosomal protein L4 (Rpl4). For each reaction we used technical duplicates, no-RT, and negative controls. The primers used for qRT-PCR are shown in Table 1.

**Table 1.** Primers used.

Gene	Forward primer	Reverse primer
Gapdh	GACATGCCGCCTGGAGAAAC	AGCCCAGGATGCCCTTTAGT
Rpl4	GCCGCTGGTGGTTGAAGATAA	CGTCGGTTTCTCATTTTGCCC
eGFP	CTACCCCGACCACATGAAGC	AAGAAGATGGTGCGCTCCTG
WPRE	GGCTGTTGGGCACTGACAAT	CCGAAGGGACGTAGCAGAAG

### Statistical analysis

A two-tailed Student t-test was used to compare each experimental group with the control group.

## Results

### AAV injection

Recombinant AAVs are the leading platform for *in vivo* delivery of gene therapies. To test the ability to transduce the rat heart with a single intraperitoneal AAV injection, we generated AAV 2/9 vectors, encoding for the green fluorescent protein eGFP under the control of the cardiac troponin T (cTnT) promoter, referred to as AAV9-cTnT-eGFP. Five -day-old rats were allocated to receive a single intraperitoneal control, ‘low dose’, or ‘high dose’ virus injection.

### Analysis of viral cardiotoxicity

**Figure 1.**
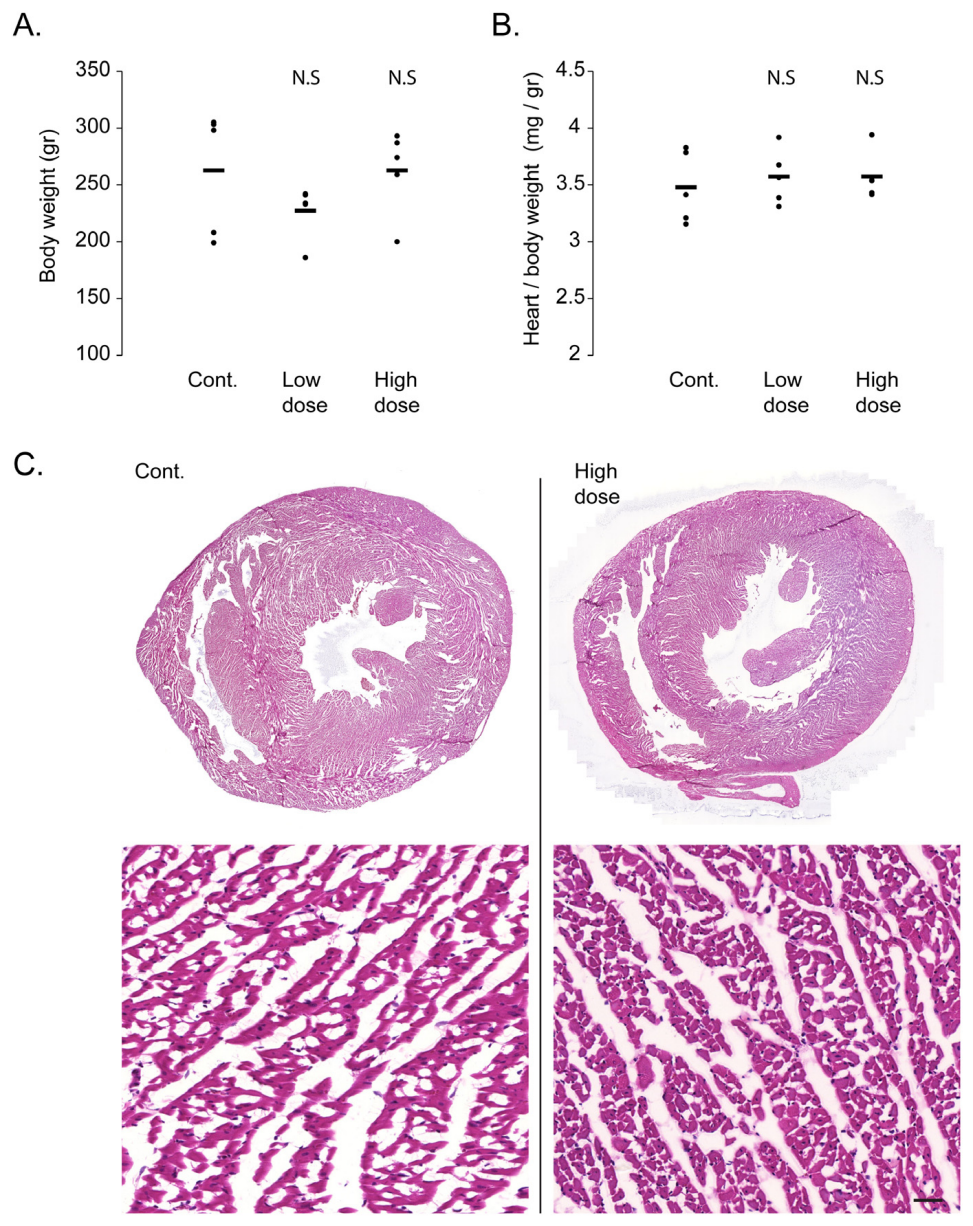
Rats injected with AAV9-cTnT-eGFP show no signs of toxicity. **A**. Gravimetric analysis show no difference in body weight between rats injected with ‘high dose’ or ‘low dose’ AAV9-cTnT-eGFP and control saline injected rats. Black bars show average and each dot represent a measurement from one rat. N.S = not statistically significant from control by Student t-test. **B**. Gravimetric analysis shows no difference in heart weight normalized to body weight between rats injected with ‘high dose’ or ‘low dose’ AAV9-cTnT-eGFP and control saline injected rats. Black bars show average and each dot represent a measurement from one rat. N.S = not statistically significant from control by Student t-test. **C**. Representative hematoxylin & eosin stained cardiac sections at low (top) and high (bottom) magnification, showing normal histology with no foci of necrosis or inflammatory cell infiltrates even in rats injected with ‘high dose’ AAV9-cTnT-eGFP (right). Control Saline injected rat heart is shown on the left. Bar = 100 µm.

One of the most promising feature of AAV as a gene therapy vector is its low toxicity ^14^. To verify that the viral transduction did not result in cardiac toxicity, we performed a gravimetric analysis of the injected rats. Bodyweight analysis did not show any significant change in either the low or high dose virus injected groups, as compared to control saline injected rats ( Figure 1A). Similarly, the heart weight, normalized to body weight, did not significantly change following viral transduction, indicating no significant cardiac atrophy or hypertrophy ( Figure 1B). To assess structural damage to the heart, necrosis, or signs of inflammation, we performed a histological analysis of the hearts with H&E staining. As shown in the representative images of control and high doses injected rat hearts ( Figure 1C), viral transduction did not result in area of necrosis or in inflammatory cell infiltrate in the heart.

**Figure 2.**
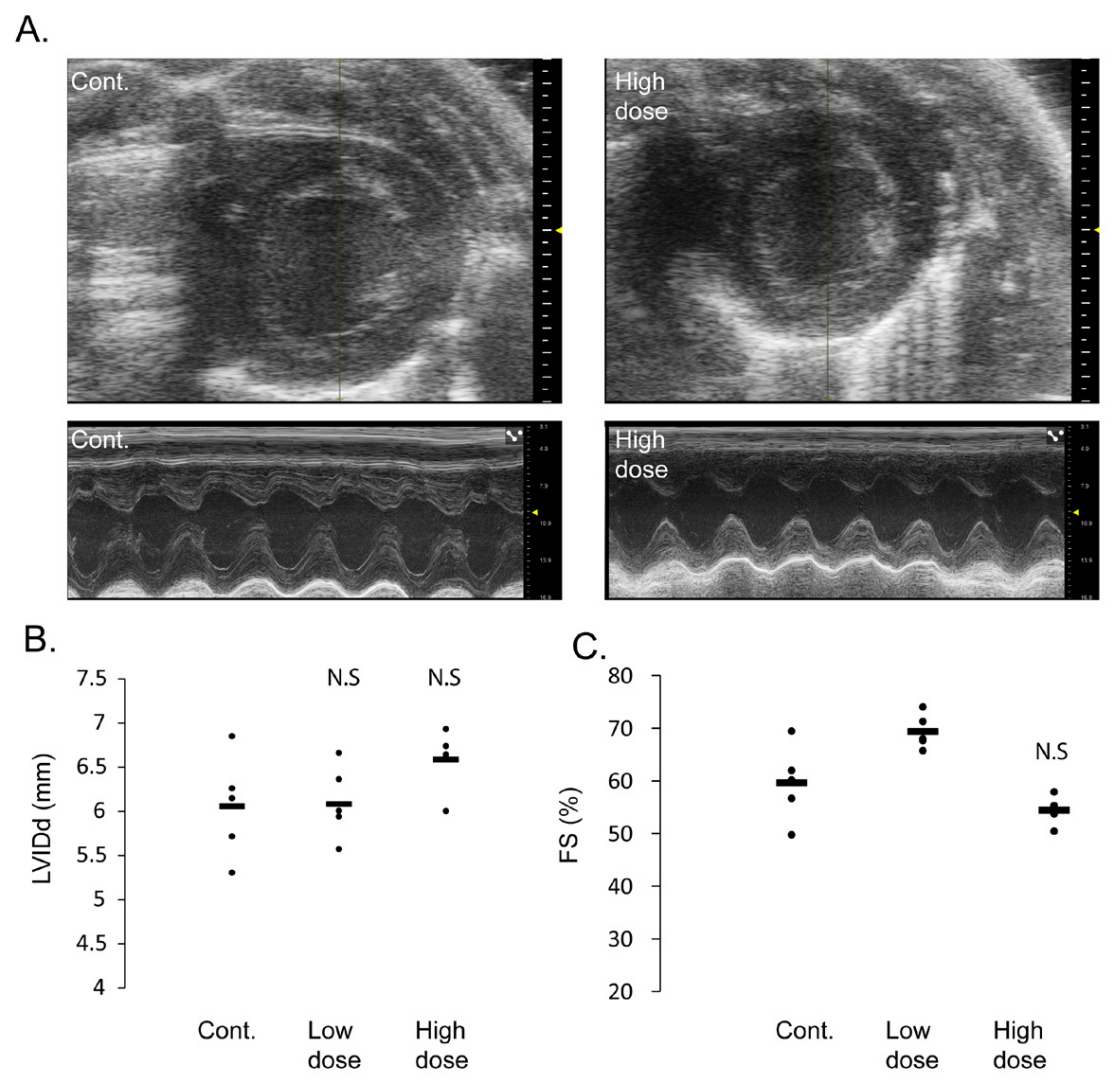
Rats injected with AAV9-cTnT-eGFP have normal cardiac dimensions and function. **A**. Representative echocardiographic images in two-dimensional short axis view (top) and M-mode (bottom) magnification, showing normal cardiac dimensions, wall thickness and contractile function even in rats injected with ‘high dose’ AAV9-cTnT-eGFP (right). Control saline injected rat heart is shown on the left. **B**. Echocardiographic measurements of left ventricular internal diameter in end diastole (LVIDd) showing no significant changes between rats injected with ‘high dose’ or ‘low dose’ AAV9-cTnT-eGFP and control saline injected rats. Black bars show average and each dot represent measurement from one rat. N.S = not statistically significant from control by Student t-test. **C**. Echocardiographic measurements of left ventricular fractional shortening (FS%) showing no decrement in cardiac function between rats injected with ‘high dose’ or ‘low dose’ AAV9-cTnT-eGFP and control saline injected rats. Black bars show average and each dot represent measurement from one rat. N.S = not statistically significant from control by Student t test.

To assess any function impairment, we performed two-dimensional echocardiography as well as M-mode measurements in all the rats. This echocardiographic analysis showed that neither the low nor the high dose injected rats had cardiac dimensions and contractile function that significantly differed from the control saline injected rats. Specifically, the left ventricular end diastolic dimeter (LVIDd) and the fractional shortening percent (FS%) were not-significantly changed in the low or high dose injected groups, as compared with the control saline injected rats ( Figure 2).

Together these data show that cardiac transduction with a single intraperitoneal injection of AAV9 in a dose of up to 1.3×10 ^12^ viral genomes does not result in any significant cardiotoxicity, cardiac atrophy, cardiac hypertrophy, or functional impairment. This approach may, therefore, be useful for cardiac studies in the rat.

### Analysis of viral transduction efficiency

**Figure 3.**
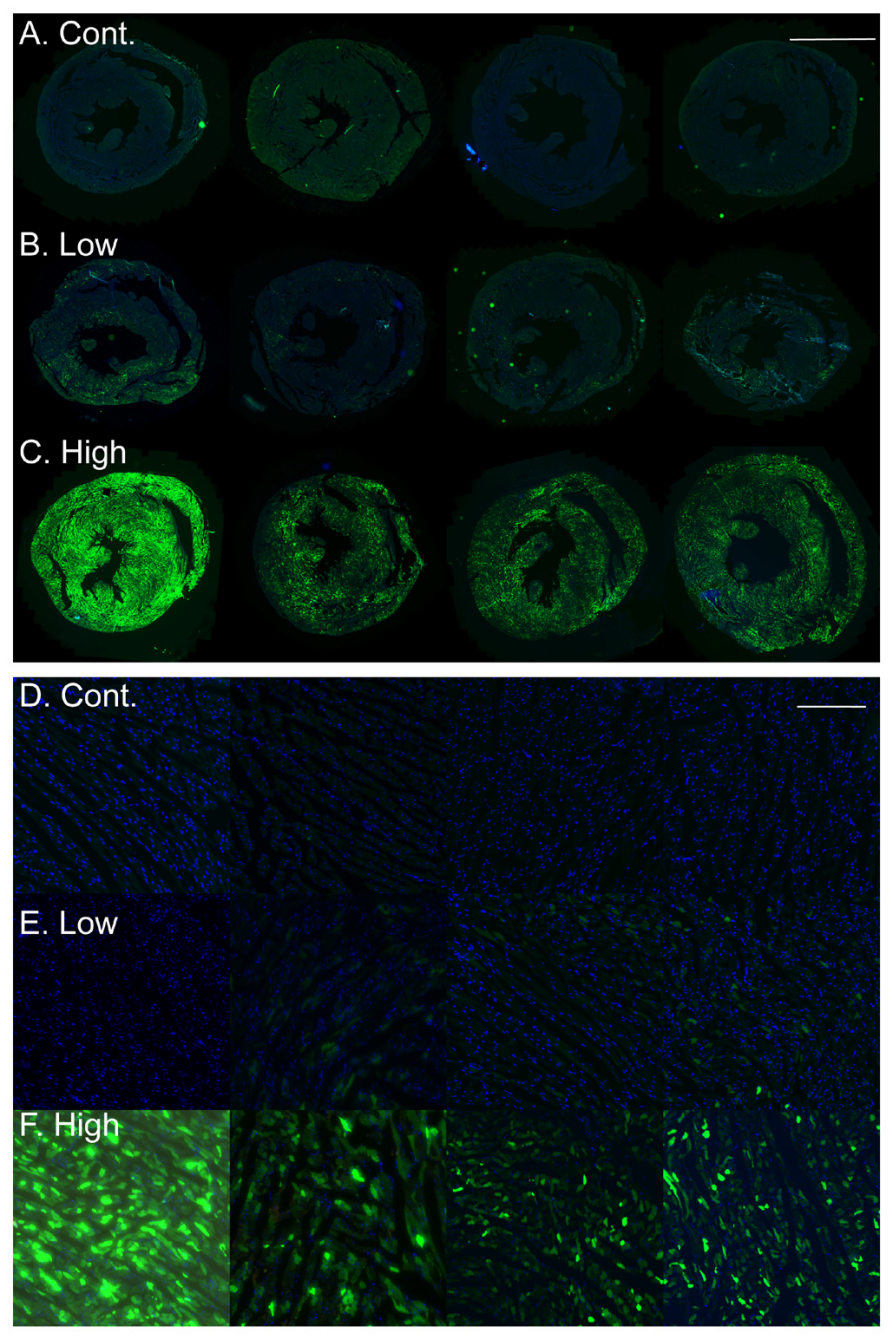
High cardiac expression of GFP in adult rats injected with AAV9-cTnT-eGFP. **A**– **C**. Low magnification images of cardiac sections of rats with GFP fluorescence (green) and DAPI nuclear stain (blue) showing no green GFP fluorescence in control saline injected rats ( **A**), few transduced GFP positive cardiomyocytes in the low dose AAV9-cTnT-eGFP injected rats ( **B**), and robust high GFP expression in the high dose AAV9-cTnT-eGFP injected rats ( **C**). Each image was taken from a different rat. Bar = 5 mm. **D**– **F**. High magnification images from the same rats shown in **A**– **C**, showing no green GFP fluorescence in control saline injected rats ( **D**), few transduced GFP positive cardiomyocytes in the low dose AAV9-cTnT-eGFP injected rats ( **E**), and robust high GFP expression in the high dose AAV9-cTnT-eGFP injected rats ( **F**). Each image was taken from a different rat. Bar = 200 µm.

AAVs can achieve high transduction efficiency *in vivo*. To assess the efficiency of our simplified approach, we analyzed cardiac sections from the control and transduced rats for eGFP green fluorescence. As can be seen in the images ( Figure 3A–F) the transduction with the low dose of AAV9-cTnT-eGFP resulted in only a low number of GFP positive cells in the heart. In contrast, transduction with the high AAV9-cTnT-eGFP dose resulted in robust and high GFP expression in the entire heart, including both the right and left ventricles in all animals, as compared with control, saline injected rat hearts, that showed no GFP green fluorescent signal.

**Figure 4.**
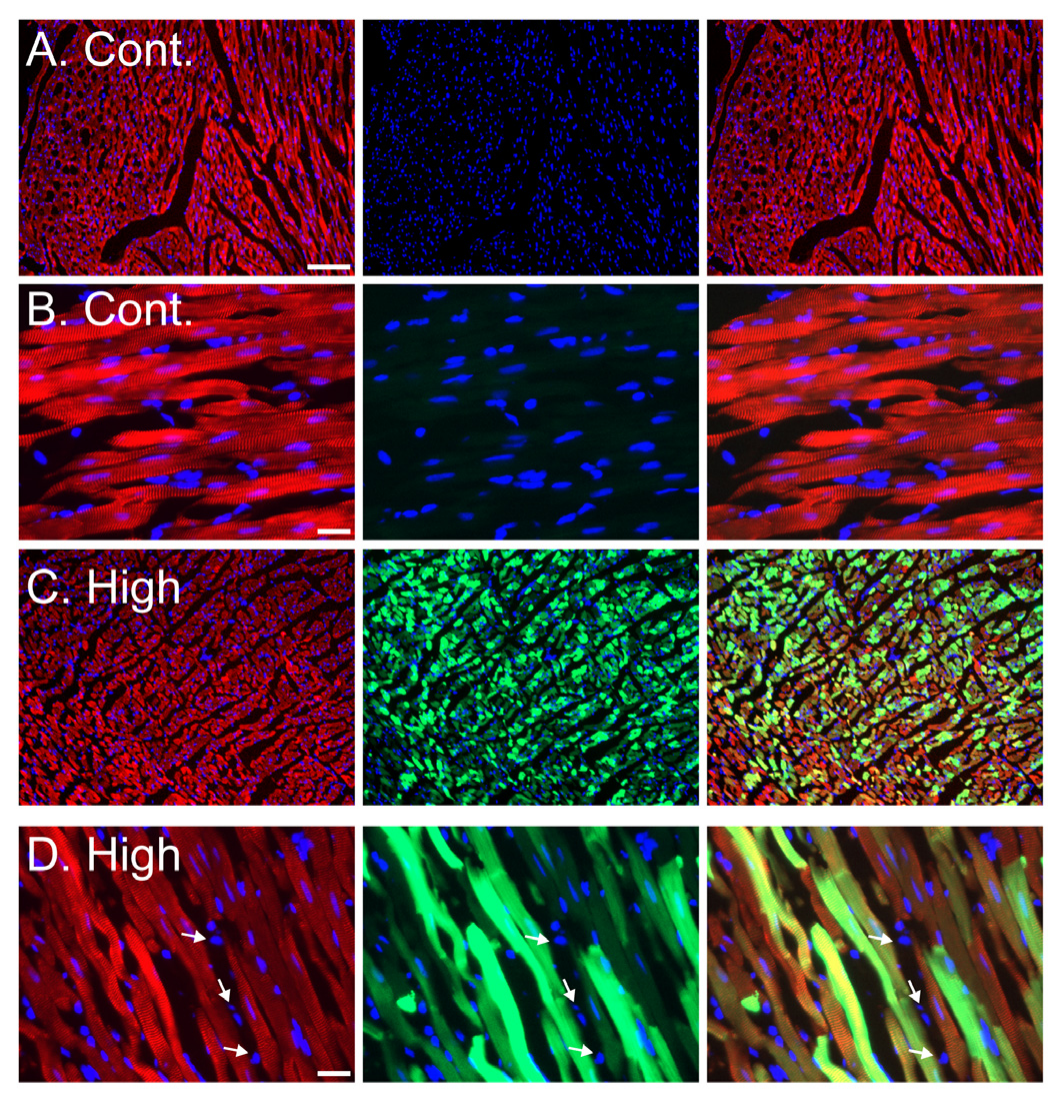
Cardiomyocyte specific expression of GFP in adult rats injected with AAV9-cTnT-eGFP. **A**– **B**. Representative images of cardiac sections of control rats ( **A** – low magnification, Bar= 100 µm; **B**- High magnification Bar= 20 µm). **C**– **D**. Representative images of cardiac sections of ‘high dose’ AAV injected rats ( **C** – low magnification, Bar= 100 µm; **D**- High magnification Bar= 20 µm). For all panels ( **A**– **D**) staining with sarcomeric α actinin immunostaining (red), eGFP fluorescence (green) and DAPI nuclear stain (blue). Left column of panels shows a composite of actinin (red) and DAPI (blue) channels, middle column shows a composite of GFP (green) and DAPI (blue) channels, and right column of panels shows a composite of all three actinin (red), GFP (green) and DAPI (blue) channels. No green GFP fluorescence is seen in cardiomyocytes from control saline injected rats ( **A**– **B**), and ~100% of cardiomyocytes show green GFP fluorescence in the high dose AAV9-cTnT-eGFP injected rats ( **C**– **D**). Importantly, non-cardiomyocyte cells, likely cardiac fibroblasts, identified by the lack of sarcomeric α actinin immunostaining do not show GFP fluorescence even in the high dose AAV9-cTnT-eGFP injected rats, demonstrating the expression is restricted to cardiomyocytes ( **D**, arrows). Bar = 20 µm.

To ensure that the bright green fluorescent signal was originating from the transduced cardiomyocytes in the heart, we performed a higher magnification analysis coupled with sarcomeric α actinin fluorescent immunostaining, to label the cardiomyocytes. As shown in the representative images ( Figure 4A–D), transduction with the high dose of AAV9-cTnT-eGFP resulted in green GFP fluorescence in almost all the cardiomyocytes. Importantly non-cardiomyocytes cells in the heart were not labeled by eGFP ( Figure 4D).

**Figure 5.**
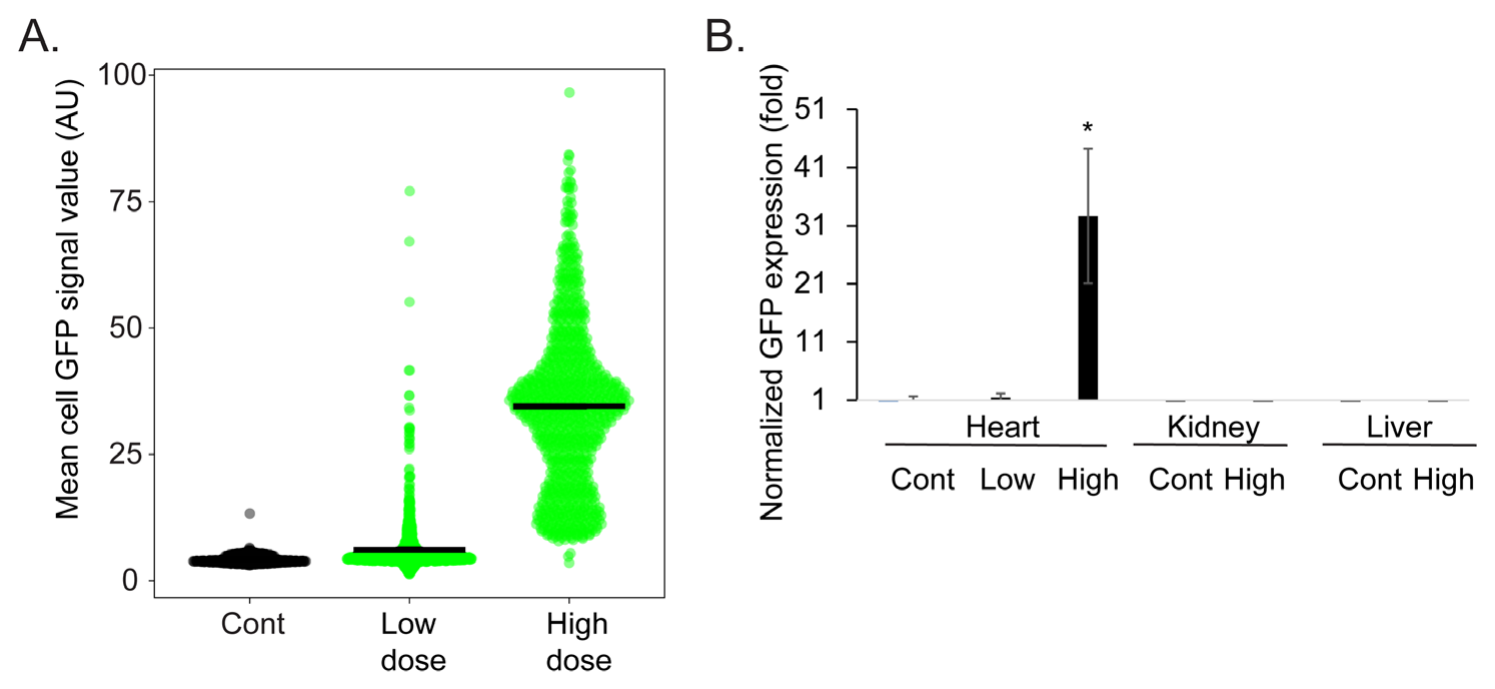
Quantification shows high transduction efficiency. **A**. A violin scatter plot of mean cardiomyocyte GFP intensity measured from random high power magnification of cardiac sections. Each point represents measurements from one cardiomyocytes in the control, low dose, and high dose AAV9-cTnT-eGFP injected rats, black bars show the mean intensity (N=3 rats, n=~900 cardiomyocytes, in each group). **B**. Quantitative reverse transcriptase PCR of normalized GFP expression showing very modest expression in the low dose AAV9-cTnT-eGFP rat hearts and high expression in the high dose AAV9-cTnT-eGFP rat hearts. No GFP expression was detectable in the liver or kidney even in the high dose AAV9-cTnT-eGFP rats. N=5 rats. Bars show the average ± standard error. * Student t-test vs. control p<0.05.

Finally, we quantified the transduction efficiency. We measured the mean GFP signal in individual cardiomyocytes chosen from random high power fields ( Figure 5A). This analysis showed a cardiomyocyte mean ± standard deviation GFP intensity of 4.24±0.71, 6.14±5.48, and 34.53±16.15 in arbitrary units in the control, low dose, and high dose injected hearts respectively (N=3 rats, n=~900 cardiomyocytes, in each group). Using a value of mean + three standard deviations of the signal intensity in the control cardiomyocytes as the cutoff for GFP expression, this would be translated to transduction efficiency (mean ± standard deviation) of 0.25± 0.35%, 20.75±7.53%, and 99.66±0.28% of the cardiomyocytes in the control, low dose, and high dose injected hearts respectively. Next, we quantified GFP mRNA expression levels using quantitative reverse transcriptase polymerase chain reaction (qRT-PCR). This analysis showed that cardiac GFP expression achieved with the low dose AAV9-cTnT-eGFP injection was 1.47±0.68 fold higher than that of the background (saline injected animals), while the expression achieved with the high dose AAV9-cTnT-eGFP injection was 32.6±11.5 higher ( Figure 5B). AAV9 is known to efficiently transduce the heart but has a general distribution of expression throughout the body, most notably the liver ^15^. To direct the expression specifically to the cardiomyocytes in the heart, we used the cardiac troponin T promoter in our viral vectors, because of its well-documented ability to drive strong, cardiomyocyte-selective transgene expression. We therefore also quantified the expression level in two additional tissues, the kidney and liver. The qRT-PCR showed that GFP expression in the kidney or liver was undetected, even in the high dose AAV9-cTnT-eGFP injected rats ( Figure 5B).

## Discussion

Some of its special features make AAV the preferred *in vivo* gene transfer vector. It is not associated with human or rat disease, it has a wide and promiscuous tropism, it is minimally immunogenic, and has a long-lived and efficient gene transfer ability ^15^. There are several serotypes of AAV that show different tropism to target tissues. AAV9 is the most cardiotropic serotype in the mouse and rat, and provides high level and stable expression in the heart ^16^. We showed here that combining the cardiotropic feature of AAV9 with the cardiac specific activity of the cTnT promoter resulted in a high but also specific expression in cardiomyocytes. We also showed that even in the high dose group we did not see expression in non-cardiomyocytes in the heart, there were no expression in the liver, and no signs of cardiotoxicity, inflammation, or functional impairment. Therefore, our approach is both safe and efficacious, and enables a scalable expression of a transgene in the adult rat heart.

There are several delivery methods for cardiac gene transfer: one method which has been described in mouse and rat is the direct intramyocardial injection, an invasive procedure that includes left thoracotomy surgery, and requires high skills ^16, 17^. Another common delivery method is intracoronary delivery via aortic root injection. This also requires invasive surgery and the use of potentially harmful vasodilators ^6^. The intravenous injection was described in mouse models ^5, 18, 19^, but in the rat, this approach resulted in low cardiac transgene expression ^20^. The efficiency of intra-venous delivery was shown to be increased by using ultrasound-targeted microbubble destruction, but this approach required continuous viral infusion through a centrally placed venous catheter and appropriate ultrasound equipment ^20^. Compared with these methods an intraperitoneal injection is simple, does not require special expertise or equipment, is safe for the animal, and does not elicit considerable stress. Intraperitoneal injection of AAV8 vectors was shown to be effective for cardiac transduction in the mouse ^21^. Neonatal gene transfer has some advantages from an immunological point of view since neonates have an immature immune system and inoculation at this period has shown to induce tolerance to the transgene products ^22^, and relatively lower vector doses are needed. Here we show for the first time, to the best of our knowledge, that a single intraperitoneal injection of AAV9 based vectors in neonatal rats is sufficient to achieve a near complete and long-lasting transduction of the adult rat heart. The ‘low dose’ used in our study of 2x10 ^11^ viral genomes is similar to the dose used in mice ^21^, however this dose resulted in low percentage of transduced cardiomyocytes in rats. In contrast, the ‘high dose’ of 1.3×10 ^12^ viral genomes was sufficient for a near complete transduction. Defining this range will allow future researchers to titrate the AAV dose to the desired level of transgene expression.

The use of AAVs is not without disadvantages. A major limitation of using AAV vectors is the relatively small transgene size (~ 4.7 kilobases) that can be cloned to the virus backbone; therefore, our approach cannot be used effectively for the expression of large genes. Recombinant AAV constructs in which the transgene does not encode a potentially tumorigenic gene product or a toxin molecule and is produced in the absence of a helper virus can usually be handled in a Biosafety Level 1 facility, but otherwise, a Biosafety Level 2 or higher may be required.

The targeting of animal genomes to add, remove, or substitute coding or non-coding sequences has revolutionized cardiovascular research. The development of the CRISPR technology has further facilitated and expanded these tools ^23^. This approach has already been utilized in the rat ^24^, but has not gained a wide-spread use as in the mouse, and generation of gene modified rats remains a difficult, time- and resources- consuming endeavor. Here we showed that a single intraperitoneal injection of AAV9 vectors encoding a transgene under the control of the cTnT promoter to neonatal rats resulted in a highly robust and highly specific cardiomyocyte transgene expression in the adult rat heart, with no signs of cardiotoxicity. In the future, this approach could be expanded to deliver Cas9 and gRNAs ^25^, or to deliver small hairpin (sh)RNAs or artificial microRNA (amiRNAs) ^26^ by AAVs to also achieve gene knockouts and knockdown in the rat heart.

## Data availability

### Underlying data

Figshare: A simple Adeno Associated Virus (AAV) -Based Approach for The Generation of Cardiac Genetic Models in Rats_ Fig 1, https://doi.org/10.6084/m9.figshare.13148633.v2 ^27^

This project contains the following underlying data:

- Raw histological images as shown in Figure 1C in TIF format

Figshare: A simple Adeno Associated Virus (AAV) -Based Approach for The Generation of Cardiac Genetic Models in Rats_figure 2, https://doi.org/10.6084/m9.figshare.13259624.v1 ^28^

This project contains the following underlying data:

- Raw echocardiographic images as shown in Figure 2A in JPG format

Figshare: A simple Adeno Associated Virus (AAV) -Based Approach for The Generation of Cardiac Genetic Models in Rats_Fig 3, https://doi.org/10.6084/m9.figshare.13148663.v1 ^29^

This project contains the following underlying data:

- Raw immunofluorescence images as shown in Figure 3 in TIF format

Figshare: A simple Adeno Associated Virus (AAV) -Based Approach for The Generation of Cardiac Genetic Models in Rats_Fig 4, https://doi.org/10.6084/m9.figshare.13148678.v2 ^30^

This project contains the following underlying data:

- Raw GFP signal images as shown in Figure 4 in TIF format

Figshare: A simple Adeno Associated Virus (AAV) -Based Approach for The Generation of Cardiac Genetic Models in Rats_gravimetry_echo_GFP fluorescence_qPCR, https://doi.org/10.6084/m9.figshare.13259366 ^31^

This project contains the following underlying data:

- gravimetric analysis.xlsx
- Echo data.xlsx
- GFP fluorescence analysis.xlsx
- qPCR raw data.xlsx

Data are available under the terms of the Creative Commons Attribution 4.0 International license (CC-BY 4.0).
