# Supplementary material for: A simple adeno-associated virus-based approach for the generation of cardiac genetic models in rats
Source: F1000Res. 2020 Dec 10;9:ISF-1441. [Version 1] doi: 10.12688/f1000research.27675.1 (PMC7863997; doi:10.12688/f1000research.27675.1)
Supplement: Peer review history

# Peer review: Reviewer response for version 1

**DOI:** 10.5256/f1000research.30591.r77191

**Published:** 2021-02-04

**Authors:** Jop H. van Berlo

**Affiliations:** 1 Lillehei Heart Institute, University of Minnesota, Minneapolis, MN, USA

The method article by Schlesinger-Laufer et al describes a simplified method for obtaining cardiomyocyte-specific expression of a gene of interest in rats. The authors provide a good rationale for why this method is useful and needed. The manuscript provides sufficient details to replicate the results presented.

 The simplified method of generating a transgenic rat involves a single intraperitoneal injection of adeno-associated viral particles that can be readily produced in cell culture or purchased from commercial sources. The authors inject the AAV particles into neonatal rats in their experiments and evaluate expression at the adult stage. The authors show that there is dose-dependent gene transduction efficiency.

 This is an important and timely report that will help investigators to implement transgenesis in their rat-based cardiovascular research.

 I only have a couple of minor comments to further improve the manuscript.

1. The methodology for measuring transduction efficiency is not very clear. Why is mean GFP intensity used as a determinant to establish a threshold for determining GFP expression? The control rat hearts should not have any GFP expression, and it makes more sense to use this as the baseline to establish threshold levels to distinguish autofluorescence from GFP expression.
2. The numbers reported are 5 animals for each group, but the text only mentions quantification on 3 animals, while the figures show 4 replicates for each group. Why this discrepancy?
3. Was any thresholding done to the images prior to measuring GFP expression?

Is the rationale for developing the new method (or application) clearly explained?

Yes

Is the description of the method technically sound?

Yes

Are the conclusions about the method and its performance adequately supported by the findings presented in the article?

Yes

If any results are presented, are all the source data underlying the results available to ensure full reproducibility?

Yes

Are sufficient details provided to allow replication of the method development and its use by others?

Yes

Reviewer Expertise:

cardiovascular physiology and pathology

I confirm that I have read this submission and believe that I have an appropriate level of expertise to confirm that it is of an acceptable scientific standard.

# Peer review: Reviewer response for version 1

**DOI:** 10.5256/f1000research.30591.r76025

**Published:** 2021-01-04

**Authors:** John Elrod, Alycia Hildebrand

**Affiliations:** 1 Center for Translational Medicine, Temple University, Philadelphia, USA; 2 Temple University, Philadelphia, PA, USA

Overview:

 The manuscript “A simple adeno-associated virus-based approach for the generation of cardiac genetic models in rats” by Kehat et al. details the development of a simplified method for the cardiac-restricted genetic expression of constructs by i.p. delivery of AAV. The rationale for the current study is the need in the scientific community to have the ability to manipulate the rat genome to better utilize the many positive characteristics =this animal model has to offer such as the ability to perform surgical procedures, and how the rat is more physiologically similar to the human and the development of newer HFpEF models etc. The study verifies the benefits of using AAVs as a gene therapy due to the low cardiac toxicity, as well as the ability to have high expression restricted to cardiomyocytes with no evidence of inflammation or functional impairment as is often associated with tamoxifen and Cre-dependent models. The authors propose that a single intraperitoneal injection of AAV9 with a cardiac troponin T promotor creates a robust cardiac specific transgenic rat model with minimal no adverse effects or extra-cardiac expression. The manuscript is well written and without any major flaws.

 Minor Concerns:

1. The manuscript would benefit by including one schematic at the beginning showing the timeline of AAV delivery and when physiological and experimental assessment was performed.
2. What is the quantification of GFP transduction for all of the hearts in Fig3B and Fig3C? In Fig5, the authors quantify n=3 in each group, however in figure 3, n=4 and in the methods n=5. Please clarify.
3. What are the authors' thoughts on why Fig3C has 1 of the 4 mice with more intense GFP transduction? Is this due to different imaging setting due to background or something?
4. The authors did not discuss direct cardiac injection at day 1 or day 2, which does not require open heart surgery. What are your thoughts on this method as a way to increase efficacy while decreasing the viral load required?

Is the rationale for developing the new method (or application) clearly explained?

Yes

Is the description of the method technically sound?

Yes

Are the conclusions about the method and its performance adequately supported by the findings presented in the article?

Yes

If any results are presented, are all the source data underlying the results available to ensure full reproducibility?

Yes

Are sufficient details provided to allow replication of the method development and its use by others?

Yes

Reviewer Expertise:

Cardiac physiology, heart failure, genetic mouse models, metabolism, mitochondria, calcium signaling

We confirm that we have read this submission and believe that we have an appropriate level of expertise to confirm that it is of an acceptable scientific standard.
